# Changes in 3-, 2-Monochloropropandiol and Glycidyl Esters during a Conventional Baking System with Addition of Antioxidants

**DOI:** 10.3390/foods9060739

**Published:** 2020-06-04

**Authors:** Kok Ming Goh, Yu Hua Wong, Faridah Abas, Oi Ming Lai, Masni Mat Yusoff, Tai Boon Tan, Yonghua Wang, Imeddedine Arbi Nehdi, Chin Ping Tan

**Affiliations:** 1 Department of Food Technology, Faculty of Food Science and Technology, Universiti Putra Malaysia, Serdang 43400, Selangor, Malaysia; kokming.goh@gmail.com (K.M.G.); wongyuhua88@hotmail.com (Y.H.W.); masniyusoff@upm.edu.my (M.M.Y.); taiboon_tan@upm.edu.my (T.B.T.); 2 Guangdong Research Center of Lipid Science Applied Engineering Technology, School of Food Science and Engineering, South China University of Technology, Guangzhou 510640, China; yonghw@scut.edu.cn; 3 Department of Food Science, Faculty of Food Science and Technology, Universiti Putra Malaysia, Serdang 43400, Selangor, Malaysia; faridah_abas@upm.edu.my; 4 Department of Bioprocess Technology, Faculty of Biotechnology and Biomolecular Sciences, Universiti Putra Malaysia, Serdang 43400, Selangor, Malaysia; omlai@upm.edu.my; 5 Chemistry Department, College of Science, King Saud University, P.O. BOX 2455, Riyadh 11451, Saudi Arabia; imed12002@gmail.com; 6 Chemistry Department, El Manar Preparatory Institute for Engineering Studies, Tunis El Manar University, P.O. Box 244, Tunis 2092, Tunisia

**Keywords:** radical, palm-based shortening, baking system, MCPD esters mitigation, GE mitigation

## Abstract

Shortening derived from palm oil is widely used in baking applications. However, palm oil and the related products are reported to contain high levels of monochloropropandiol (MCPD) ester and glycidyl ester (GE). MCPD and glycidol are known as process contaminants, which are carcinogenic and genotoxic compounds, respectively. The objective was to evaluate the effects of antioxidant addition in palm olein and stearin to the content of MCPD esters and GE in baked cake. Butylated hydroxyanisole (BHA), rosemary extract and tocopherol were used to fortify the samples at 200 mg/kg and in combinations (400, 600 and 800 mg/kg rosemary or tocopherol combined with 200 mg/kg BHA). The MCPD esters and GE content, radical formation and the quality of the fats portion were analyzed. The results showed that palm olein fortified with rosemary extract yielded less 2-MCPD ester. The GE content was lower when soft stearin was fortified with rosemary. ESR spectrometry measurements showed that the antioxidants were effective to reduce radical formation. The synergistic effects of combining antioxidants controlled the contaminants formation. In conclusion, oxidation stability was comparable either in the single or combined antioxidants. Tocopherol in combination with BHA was more effective in controlling the MCPD esters and GE formation.

## 1. Introduction

Palm oil and the derivative products from palm oil are well-recognized as an important raw material in the food industry [1]. Palm oil is unique due to the balance in its saturated and unsaturated fatty acid composition [2]. Each fraction derived from palm oil serves a specific function in the food industry [3]. Fractionation of palm oil yields olein and stearin fractions. The olein fraction is used as a frying medium due to its remarkable oxidation stability. On the other hand, stearin (solid fraction) provides consistency, portability and texture to the products [4].

Palm oil in the baking industry functions as a shortening. A shortening is a pure fat product containing animal and/or vegetable oils that is formulated and processed according to functionality [3]. The stearin fraction of palm oil performs as a good shortening due to its high solid fat content (SFC) and β’ polymorphic form [5]. In addition, a recent study showed that the application of soft fraction of palm oil can deliver a higher volume in baked cakes [6]. Generally, a highly functional specialty fat is achievable by blending, hydrogenation, and interesterification process [7].

Generally, palm oil and fats are known to contain higher monochloropropandiol (MCPD) esters and glycidyl esters (GE) compared to other types of refined vegetable oils [8]. Furthermore, 2- and 3-MCPD esters are heat-induced food contaminants, and they are carcinogenic [9]. In addition, GE is often considered a coexisting contaminant with MCPD esters in refined vegetable oils and is shown to be genotoxic in animal studies [10].

Furthermore, MCPD esters and/or GE can be induced by processing under suitable conditions. Water, salt, glycerol or lipid precursors undergoing thermal processing can trigger the formation of MCPD esters [11]. Studies have reported that glycerol can act as a strong precursor to form the 3-MCPD ester on a weight basis [12]. Other important precursors, such as monoacylglycerol (MAG), diacylglycerol (DAG) and triacylglycerol (TAG), also play roles in the formation of MCPD esters [13]. Four MCPD ester formation pathways were reported by Rahn and Yaylayan, which are directly responsible for the nucleophilic attack at the glycerol carbon carrying an ester group or a hydroxyl group, formation of the acyloxonium ion and formation of the glycidol ester with an epoxide ring [14]. Apart from these well-known mechanisms, 3-MCPD ester (di-ester form) is believed to be formed using a DAG undergoing free radical reaction mechanisms [15].

Since the generation of free radicals is also related to heating processes, investigation of the effects of antioxidant on the changes in MCPD esters and GE during conventional baking using palm-based shortening is the main focus of this study. Antioxidants used in food applications can extend the fatty food storage time by slowing down the oxidation process [16] For example, butylated hydroxyanisole (BHA) is used in fried food, and the application of two or more antioxidants can act synergistically to maximize the functional properties [17,18]. It is also reported that antioxidant extracts incorporated into the food or food packaging material can hinder the lipid deterioration within a meat product [19,20]. Generally, the antioxidants prevent the generation of free radical (e.g., hydroxyl radicals) by acting as a hydrogen or electron donor [21,22]. Additionally, a previous study of the changes in 3-MCPD ester and GE content of a frying system suggested addition of antioxidants to the palm olein was effective to reduce the level of MCPD ester and GE contaminations [23]. As MCPD ester formation can be triggered via a radical mechanism [24], reducing radical formations by antioxidant fortification in baking recipes is a potential mitigation tool that is a straightforward and low-cost alternative.

Indeed, the maximum level of GE has been set at 1 mg/kg for vegetables fats and oils as final consumers oil or as food ingredient. A more stringent limit, which is 0.5 mg/kg of GE, is allowed when the vegetable fats and oils are destined for baby food. At the same time, the European Commission has suggested the maximum level of 3-MCPD esters of palm oil is 2.5 mg/kg and the limit from a vegetable oil derived from coconut, maize, rapeseed, sunflower, soybean and palm kernel oil should not have 3-MCPD esters of more than 1.25 mg/kg. Meanwhile, the tolerable daily intake (TDI) of 3-MCPD is 2 µg/kg body weight [25,26].

In this study, palm olein and soft stearin were chosen as the shortening in a recipe based on an evaluation study of quality characteristics attributed to the finished products in our laboratory [6]. The shortening was fortified with BHA, rosemary extract and tocopherol, either in a single dosage or in combination with natural and synthetic antioxidants. Effects of different antioxidants on the changes in the radicals present in the samples, MCPD esters, GE, oxidation stage (specific extinction values) and acylglycerol composition of the fats portion were studied.

## 2. Materials and Methods 

### 2.1. Materials and Chemicals

All the baking ingredients were purchased from a local supplier (JC Rainbow, Selangor, Malaysia), except the whole egg powder was obtained from AgroEgg (Singapore). Palm olein and soft stearin were donated by a local palm oil refinery company. Baking materials were kept in an air-tight container at room temperature, while the egg powder and fats portion were kept under a chilled temperature (4 °C) until used. All the chemicals were purchased from a local supplier (Merck, Selangor, Malaysia). MCPD esters and GE standards were purchased from Toronto Research Chemicals, Inc. (North York, ON, Canada). All chemicals were conserved in suitable condition according to the material safety data sheet (MSDS) while the MCPD ester and GE standards were kept in amber bottles at −20 °C prior to the analysis. 

### 2.2. Fortification of Palm Olein and Soft Stearin with Antioxidants

Each of the antioxidants, BHA (butylated hydroxyanisole, 96%, Fisher Scientific Acros, Pittsburgh, PA 15275, USA), rosemary extract (Oleoresin Rosemary, Herbalox brand, Type O) and tocopherol (DL-α-Tocopherol, 99%, Merck, Selangor, Malaysia), was measured at the weight needed and added to the palm olein or stearin to make a final concentration of antioxidants in the oil of 200 mg/kg. The fats and antioxidants were placed in a 1000-mL beaker and heated over a hotplate stirrer, stirred at 200 rpm, at 50 °C for 10 min, to homogenize the sample. A similar procedure was applied with different concentrations of the natural antioxidants (400, 800 and 1200 mg/kg) and a fixed amount of BHA (200 mg/kg). 

### 2.3. Sample Preparation

The cake samples were prepared based on a modified Madeira cake recipe from a previous study. The recipe was measured in weight basis instead of percentage to fix the number of the sample size during each preparation. Each portion of the recipe was made up of 40 g fat fortified with the antioxidant, 120 g wheat flour, 50 g granulated sugar, 100 g egg powder mix (ratio of egg powder to water, 1:3) and 5 g leavening agent (sodium bicarbonate) [27]. 

The preparation began with mixing the fats and granulated sugar using a kitchen mixer for 10 min until homogenized. Whole egg powder was mixed with water at given ratio, and it was added to mix for another 5 min. Finally, the flour and baking powder were added together. The mixing was continued for another 5 min. The speed of the kitchen mixer was maintained at medium speed throughout the mixing processes. The well-mixed batter was distributed into a 5 cm × 5 cm × 5 cm paper cup at 40 ± 1 g. Each bake in the oven produced 25 pieces of cake in the middle tray. The baking temperature was fixed at 160 °C, and the baking duration was 20 min throughout the sample preparation. The described design during baking was adapted from a previous study in order to stabilize the heat distribution during each batch of the baking process [6]. 

### 2.4. Fat Extraction

The fats portion from the cake sample were extracted using a fat extraction system (Soxtec^TM^ 8000, Foss, Hilleroed, Denmark). The sample was pre-dried in an oven for 24 h at 98 °C. Every 2 g of sample was extracted with 80 mL petroleum ether (boiling point 40–60 °C, AR grade, Merck, Selangor, Malaysia) as the extraction solvent. 

### 2.5. Free Fatty Acid Analysis (Acidity Value)

The free fatty acid content was determined by a modified AOCS Official Method Ca 5a-40. Samples were titrated against alkaline (NaOH) to the phenolphthalein end-point. Every 2 g of test sample was diluted with 10 mL of hot neutralized alcohol (95% ethanol) and 0.5 mL of phenolphthalein indicator. The normality of the NaOH solution was 0.0005 M. The FFA content was expressed as the palmitic acid equivalent in percentage per gram of oil sample [28,29].

### 2.6. Electron Spin Resonance Measurements

The electron spin resonance (ESR) spectral analysis and G-value measurements were performed using an ESR spectrometer (JES-FA 2000, JEOL, Tokyo, Japan). A sample portion of 10 mg was placed in a quartz sample tube. To visualize the free radical activities, the measurement conditions of the first derivative spectrum used a 9181.6 MHz microwave frequency at 1 mW power, 1.2 mT modulation amplitude, 0.03 s time constant and 60 s sweep time. The free radical was compared to the signal intensity, expressed in an arbitrary unit per unit sample weight (AU/mg).

### 2.7. Total Chlorine Analysis

The total chlorine was analyzed using energy dispersive X-ray (EDX) fluorescence spectrometry (EDX-8000, Shimadzu, Kyoto, Japan). Briefly, a 2 g dried cake sample was lightly crushed and placed into a sample holder (31 mm X-Cell) and covered by an EDX film (3525 Ultralene, 4 µm thickness). Then, the cells were placed on the sample tray for measurement. Measurement was started by internal calibration against a blank sample. Then, samples were measured and rotated automatically according to the sample tray position. The content of total chlorine analyzed will be listed as its percentage.

### 2.8. Oxidation of the Fats Portion

A portion of the 20 mg oil sample was measured and diluted in 100 mL of isooctane. An absorbance value of the specific extinction values of 232 nm and 268 nm was measured as described in the AOCS Official Method, Ch. 5-91.

### 2.9. Aclyglycerol Composition

The FFA and monoacylglycerol (MAG) to DAG ratio as well as the 1,3-DAG to 1,2-DAG ratio were analyzed according to the AOCS Official Method Cd 11-96 using an HPLC system (Alliance model Waters 22.695 Separation Modules, Wilmslow, UK) equipped with an ELSD detector (Alliance model Waters 2424 ELS Detector, Wilmslow, UK).

The test sample was diluted in a mixture of *n*-hexane and isopropanol (ratio 9:1). The diluted sample was filtered through a PTFE membrane filter and injected into a normal phase silica column (150 mm × 4.6 mm, LiChrospher ^®^ 60 Si (10 µm), Hibar, Merck, Selangor, Malaysia). The injection volume was 20 µL. Eluent A (*n*-hexane) and eluent B (*n*-hexane:ethyl acetate:isopropanol at an 8:1:1 ratio) were used to establish a gradient program. The gradient started with 98% of eluent A and 2% of eluent B and reached 65% eluent A and 35% of eluent B at 8 min; then, 2% of eluent A and 98% of eluent B at 8.5 min, held for 15 min; and lastly, 98% of eluent A and 2% of eluent B at 15.1 min, held until 19 min. Total flow rate of the eluent was 2 mL/min. The column oven temperature was 40 °C, while the drift tube and temperature of the detector was 60 and 36 °C, respectively. Nitrogen gas flow was constant at 40 psi.

### 2.10. MCPD Esters and GE Analysis

Determination of the 2-MCPD ester, 3-MCPD ester and GE was carried out by using an indirect quantification method according to the AOCS Official Method Cd 29a-13. All the sample preparation procedures were carried out as described in the manual. A derivatized sample was injected into a gas chromatograph with a triple quadrupole mass spectrometer detector (Shimadzu, GCMS-TQ 8040, Kyoto, Japan). The sample injection amount was 1 µL. Separation was performed using a capillary column Equity-1MS (Supelco, 30 m length, 0.25 mm i.d., and 1.0 μm film thickness, Taufkirchen, Germany). The transfer line temperature was 300 °C, followed by an increasing temperature program (80 °C at the start, held for 1 min; 10 °C/min until 170 °C; 3 °C/min until 200 °C; and then 15 °C/min until 300 °C, held for 15 min). Multiple reaction monitoring (MRM) was used to detect the presence of MCPD esters and GE. Each compound was identified and quantified based on the targeted ion and reference ion. Targeted ion pairs of 3-MCPD-d5, 3-MCPD and 2-MCPD were *m*/*z* 150.00 > 93.10, 147 > 91.10 and 196 > 104.10, respectively. Meanwhile, targeted ion pairs of 3-MBPD-d5 and 3-MBPD (GE) were 150 > 93.10 and 242.00 > 147.10, respectively [30].

### 2.11. Statistical Analysis

Data are presented as the mean value with the standard deviation. Calculation of the mean values, standard deviations and statistical analyses were computed using MINITAB 17 software (Minitab Inc., State College, PA, USA). Significance among the mean values of the data was tested by one-way analysis of variance (ANOVA) with Tukey’s test at a confidence level of 95% (α = 0.05). 

## 3. Results and Discussion

### 3.1. Effects of Different Antioxidants on the Changes of MCPD Esters and GE

The concentration of the raw samples (without antioxidants) were checked prior to the experiments. The 3-MCPD ester content of palm olein and soft stearin was between 3.15 and 4.19 mg/kg and 2.38 and 2.59 mg/kg, respectively. The content of 2-MCPD esters of palm olein and soft stearin was about half of the 3-MCPD ester, which was recorded for palm olein and soft stearin sample as between 1.77 and 2.11 mg/kg and 1.29 and 1.40 mg/kg, respectively. Lastly, the GE content of the palm olein sample was between 5.82 and 7.05 mg/kg, while a soft stearin sample contained GE at a concentration between 2.82 and 3.12 mg/kg. These findings are in agreement with previous findings in palm olein samples for frying [31], margarine samples from the market [30] and a market survey on palm oil samples from different countries [32]. GE is commonly higher than the content of 3-MCPD esters, as stated in the EFSA journal in 2016 [8]. The experiment was continued by analyzing the effects of an antioxidant at a fixed concentration (200 mg/kg) on the changes of MCPD esters and GE content after baking at 160 °C for 20 min using palm olein or soft stearin as the shortening. From Table 1, the content of the 2-MCPD ester found in palm olein fortified with 200 mg/kg rosemary extract and tocopherol was significantly lower compared to BHA. In addition, in the soft stearin sample, the rosemary extract-fortified sample yielded a lower GE content at 0.607 mg/kg. Overall, the content of 2- and 3-MCPD ester was found slightly lower when rosemary extract and tocopherol were used compared to BHA.

### 3.2. Electron Spin Resonance Measurement

**Figure 1:**
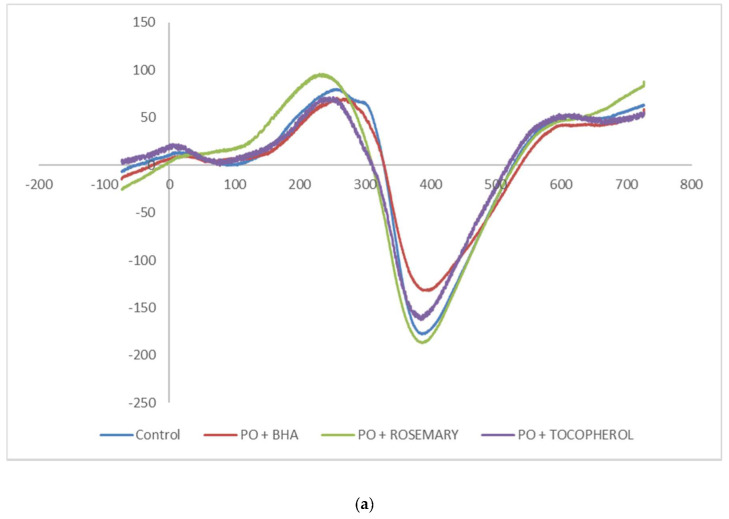

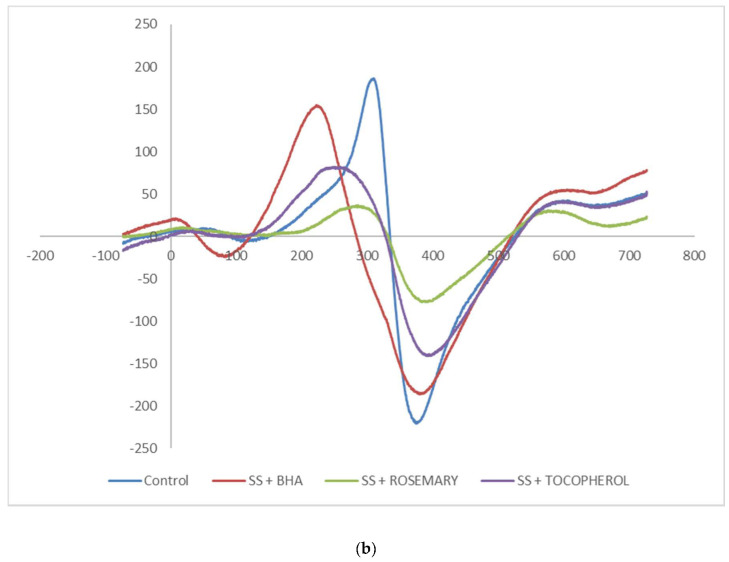
(**a**) The ESR spectra of a palm olein sample fortified with BHA, rosemary extract and tocopherol at 200 mg/kg and the control (no antioxidant); (**b**) the ESR spectra of a soft stearin sample fortified with BHA, rosemary extract and tocopherol at 200 mg/kg and the control (no antioxidant).

The Electron Spin Resonance (ESR) spectra as shown in Figure 1a,b are palm olein and soft stearin samples, respectively. ESR can measure the chemical species with unpaired electron(s), and they are normally referring to, but not limited to, the free radicals and transition metal ions [33]. From Figure 1a, the results showed similar ESR spectra among the palm olein samples. The signal intensity from the highest to lowest was rosemary ≈ control (without antioxidant added) > tocopherol > BHA. However, adding antioxidants to the soft stearin sample yielded different results. From Figure 1b, the ESR signal intensity was highest in the control sample, indicating significant amounts of radicals were present, followed in the signal intensity sequence by BHA > tocopherol > rosemary.

The results clearly showed that addition of antioxidants to the baking recipe was effective to reduce the radical presence in the sample. The reduced signal suggested antioxidant was able to scavenge the free radicals [34] formed during the baking process.

The G-values obtained from the ESR spectrum are listed in Table 2. The G-value is defined as the number of molecules destroyed or products formed per 100 eV of energy absorbed [35]. The G-value of a substance is normally constant, and the G-value of a free radical is 2.00. The G-values of the control samples were 1.98 and 1.96 for palm olein and soft stearin, respectively. A G-value less than 2.00 is considered an electron-poor substance. Therefore, addition of antioxidants to soft stearin was more effective to inhibit the production of radicals. Soft stearin has more saturated fatty acid than palm olein, and the saturated structures are resistant to reactions such as oxidation. 

### 3.3. Oxidation and Stability of the Fats Portion with Addition of Single Antioxidant

The fats extracted from the baked sample were further analyzed with the oxidation state (K_268_ and K_232_ value), free fatty acid (FFA) content and the ratio of acylglycerol composition to DAG. Table 2 shows that the rosemary extract was able to suppress the formation of FFA in both the palm olein and soft stearin samples during the baking process. The FFA content was between 0.24% and 0.27% of the palmitic acid equivalent. 

A previous study on antioxidant addition to frying palm olein showed that rosemary extract was able to perverse the oil quality in terms of oxidation stability [23]. Similarly, rosemary addition to the shortening was able to give a lower FFA-to-DAG ratio compared to BHA or tocopherol. In terms of the FFA-to-DAG ratio, we observed that the rosemary extract was able to inhibit the formation of FFA and/or DAG in the fats portion, followed by BHA and tocopherol. The formation of FFA and DAG was derived from TAG hydrolysis, and results showed that addition of rosemary extract at 200 mg/kg was effective to suppress the hydrolysis process among the samples studied. 

The presence of 1,3 DAG is always abundant in palm-based fats and oils because DAG is a more stable isomer than DAG with a fatty acid chain at the 1,2-positions [36]. The ratio of 1,3-DAG to 1,2-DAG among all the samples studied ranged from 2.415 to 2.599. These values were lower than the control sample. The control sample without antioxidant addition showed a 1,3- to 1,2-DAG ratio at 2.583 and 2.898, for palm olein and soft stearin, respectively. This observation showed that the antioxidant addition was not effective to maintain the DAG in its stable form, which is the 1,3-position of DAG. The results obtained from the same laboratory, with the baking system at a higher temperature (180 or 200 °C), further lowered the ratio of 1,3- to 1,2-DAG because hydrolysis of TAG yielded unstable 1,2-DAG instead of stabilized 1,3-DAG [6]. 

The oxidation state of the samples was checked by specific extinction values. The values as shown in Table 2, for samples fortified with BHA and rosemary, were able to give a close absorbance at K_268_ and K_232_. However, tocopherol was not as effective as BHA and/or rosemary to inhibit the formation of conjugated dienes (CDs) and trienes (CTs). Tocopherol had a significantly (*p* < 0.05) higher value of CT and CD observed in both tocopherol-fortified palm olein and soft stearin. 

### 3.4. Synergistic Effect of Antioxidant on the Changes in MCPD Esters and GE

**Figure 2:**
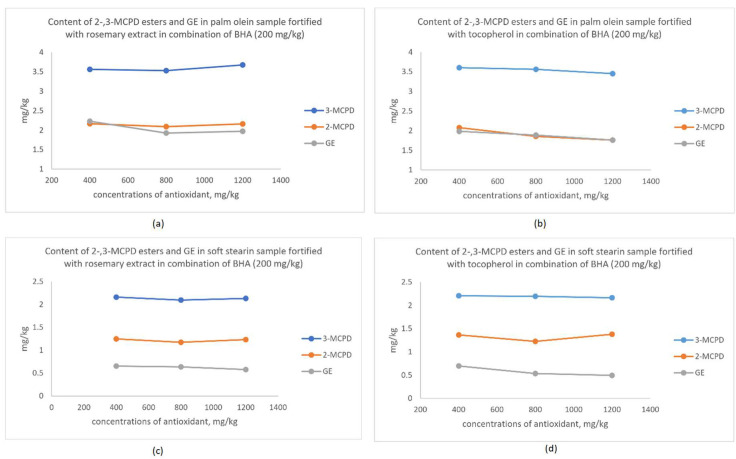
(**a**) Changes of 3- and 2-MCPD esters and GE content in a palm olein sample with different concentrations of rosemary extract and BHA (200 mg/kg). (**b**) Changes in 3- and 2-MCPD esters and GE content in a palm olein sample with different concentrations of tocopherol and BHA (200 mg/kg). (**c**) Changes of 3- and 2-MCPD esters and GE content in a soft stearin sample with different concentrations of rosemary extract and BHA (200 mg/kg). (**d**) Changes of 3- and 2-MCPD esters and GE content in a soft stearin sample with different concentrations of tocopherol and BHA (200 mg/kg).

From the discussion in the above section, MCPD esters and GE changes were not obvious when only a single dose of antioxidant was used. Therefore, the experiment was extended to fortify the shortening in combination with BHA and either rosemary extract or tocopherol. Rosemary or tocopherol was added to the shortening containing 200 mg/kg BHA at 400, 800 and 1200 mg/kg. Data obtained are shown in Figure 2.

In the palm olein sample (see Figure 2a,b), when the rosemary extract was added at concentrations of 800 and 1200 mg/kg in combination with 200 mg/kg of BHA, the GE content was able to be reduced from 2.230 mg/kg to 1.925 and 1.972 mg/kg. The reduction was statistically significant (*p* < 0.05). However, addition of tocopherol in combination with BHA was more effective in lowering MCPD esters and GE formation during the baking process. Tocopherol addition at a concentration of 800 mg/kg was sufficient to reduce the formation of 2-MCPD ester (1.854 mg/kg) and GE (1.888 mg/kg). Notably, the content of 2- and 3-MCPD esters and GE were lowered significantly (*p* < 0.05) to 1.758 mg/kg, 3.453 mg/kg and 1.761 mg/kg, respectively, when the tocopherol concentration was increased to 1200 mg/kg. 

In the soft stearin sample (see Figure 2c,d), the 3-MCPD ester content was reduced with 800 mg/kg of rosemary extract in combination with 200 mg/kg BHA. No significant changes occurred in the 2-MCPD ester and GE content. The GE content was reduced significantly (*p* < 0.05) from 0.696 mg/kg to 0.533 and 0.494 mg/kg when the addition of tocopherol was increased to 800 and 1200 mg/kg. 

Overall, the figures showed that tocopherol combined with BHA was more effective in producing a finished product with lower contaminants compared to the rosemary extract in combination with BHA. Since tocopherol alone was able to inhibit the radical formation (Figure 1), its synergistic effects with BHA was expected. Total antioxidant capacities by combining antioxidants was proven to be more effectives in scavenging the radical [37]. Therefore, the radical-induced forming mechanism of the MCPD esters and GE [15] was interrupted by the tocopherol and rosemary extract combined with BHA.

### 3.5. Synergistic Effect of Antioxidants on the Changes of Oxidation State and Stability

From Table 3, the results showed that the overall changes of the oxidation state and stability of the acylglycerol composition was stable by antioxidant synergistic effects. In terms of oxidation state, rosemary when combined with BHA was more effective in inhibiting the formation of CT. The ratio among FFA/DAG, MAG/DAG and 1,3-/1,2-DAG was very similar for each treatment. The oxidation level of the samples studied was considered at its minimal level according to the synergistic effects of rosemary or tocopherol addition combined with BHA. The FFA % by titration method showed an increased FFA % when the concentration of the antioxidant was higher, but that did not affect the specific extinction value and acylglycerol composition. In short, the data agreed with the previous findings stated that the synergism between the antioxidants works to control lipid oxidation in a Rancimat test [38] and in sunflower oil triacylglycerol [39].

### 3.6. MCPD Esters and GE Formation with the Presence of Potential Precursors

MCPD esters formation pathways are well discussed in the available literature. MCPD esters can be formed via a direct substitution of the chlorine anion by a glycerol carbon atom (replacing either the OH group or a fatty acid ester group), and via an intermediate epoxide ring or acyloxonium group before a nucleophilic attack by a chlorine anion [14]. In addition, a more recent study also suggested a chlorine radical or chlorine compound attack on a cyclic acyloxonium free radical intermediate, through the free radical mechanism [24,40].

By looking at these possible formation mechanisms described in the available literature, the baking system is a favorable system to encourage the formation of MCPD esters and/or GE. In addition, chlorine, as one of the important precursors for 3-MCPD ester formation, can be introduced through various sources of ingredients, such as flour [41]. The relationship between chlorine content and the 3-MCPD ester has proven to be a strong positive one based on a previous study [42]. However, the recipe used during the experiments was controlled and therefore no significant change in the total chlorine percentage was observed throughout the experiment (see Table 2). Therefore, it is known that the formation or inhibition of the MCPD esters by antioxidants in this study was not affected by the factor of chlorine amount. The MCPD esters are formed through multiple reaction pathways, and different pathways can occur simultaneously. Moreover, the formation and decomposition of the MCPD esters should occur simultaneously as well. For instance, the scavenging activity exhibited by the rosemary extract was positive in the soft stearin sample (ESR measurements), but the content of the 2- and 3-MCPD esters was not reduced significantly. Similarly, the presence of tocopherol in combination with BHA was able to give a finished product with lower MCPD esters, but the ability to inhibit oxidation was weaker. The observations showed that there was no direct relationship between the presence of radical as a potential precursor to the MCPD esters formation. However, due to the abundance of potential precursors (TAG, DAG and chlorine compounds), MCPD esters could still be formed via substitution of chlorine and acyloxonium intermediate reactions. There is controversial discussion about the relationship of GE and MCPD esters formation, and therefore the use of antioxidants in reducing GE is still unclear [10]. However, a recent study, concerning the addition of antioxidants to a frying system, had clearly shown that antioxidants were effective in controlling and inhibiting the formation of 3-MCPD ester and GE at the same time [23]. The results showed that GE content was lowered during a series of tests of a continuous frying system with the presence of antioxidants.

In this study, our results showed that antioxidants delay the oxidation of the lipid portion and were able to scavenge the radicals by donating electrons in the baking system either at a single dosage or in combinations with two antioxidants. A synergistic effect by combining the natural (tocopherol or rosemary extract) and synthetic (BHA) antioxidants was more effective to control the MCPD esters and GE formation, especially when a higher concentration of natural antioxidant was used (800 and/or 1200 mg/kg).

## 4. Conclusions

The addition of antioxidants into a baking recipe using palm olein and soft stearin as shortening inhibited the formation of radicals observed from the ESR spectrometry measurements. The fats portion from the finished product was considered stable among all the treatments with antioxidants. At a single dosage, rosemary extract was more effective than either tocopherol or BHA to produce lower 2-MCPD ester, 3-MCPD ester and GE in both the studied samples. In addition, a single dose of antioxidants was sufficient to improve the stability of the finished products in terms of oxidation status. A combination of rosemary extract or tocopherol with 200 mg/kg BHA was more effective in controlling the formation of 2-MCPD ester, 3-MCPD ester and GE when the concentration of the natural antioxidant was higher (800 and 1200 mg/kg).

## Figures and Tables

**Table 1:** Effects of different antioxidants (200 ppm) on the content of 2-monochloropropandiol (MCPD), 3-MCPD and glycidyl ester (GE) on the fats portion extracted from cake baked at 160 °C for 20 min with different shortenings.

Sample	Antioxidant	3-MCPD, mg/kg	2-MCPD, mg/kg	GE, mg/kg
	BHA	3.439 ± 0.029 ^a^	2.334 ± 0.021 ^a^	1.987 ± 0.039 ^a^
Palm Olein	Rosemary	3.431 ± 0.065 ^a^	2.051 ± 0.010 ^b^	1.979 ± 0.029 ^a^
	Tocopherol	3.527 ± 0.189 ^a^	2.143 ± 0.105 ^b^	1.985 ± 0.047 ^a^
	BHA	2.222 ± 0.028 ^a^	1.534 ± 0.114 ^a^	0.665 ± 0.001 ^a^
Soft Stearin	Rosemary	2.176 ± 0.032 ^a^	1.489 ± 0.040 ^a^	0.607 ± 0.032 ^b^
	Tocopherol	2.172 ± 0.023 ^a^	1.435 ± 0.012 ^a^	0.668 ± 0.010 ^a^

Values are the mean of two determinations from two replicate experiments ± standard deviation (*n* = 4). Mean values with different superscript letters in the same column are significantly different at *p* < 0.05.

**Table 2:** Effects of different antioxidants (200 ppm) on the FFA content, specific extinction value and acylglycerol composition in the fats portion extracted from cake baked at 160 °C for 20 min with different shortenings.

Sample	Antioxidant	FFA, %	K_268_, CT	K_232_, CD	FFA/DAG	MAG/DAG	1,3/1,2-DAG	G-Value	Total Chlorine, %
Palm Olein	BHA	0.39 ± 0.14 ^b^	0.757 ± 0.072 ^a^	4.393 ± 0.505 ^b^	0.202 ± 0.006 ^b^	0.018 ± 0.002 ^a^	2.599 ± 0.017 ^a^	2.000 ± 0.004 ^b^	0.182 ± 0.010 ^a^
	Rosemary	0.27 ± 0.05 ^ab^	0.753 ± 0.111 ^a^	4.149 ± 0.497 ^b^	0.172 ± 0.005 ^c^	0.018 ± 0.004 ^a^	2.583 ± 0.053 ^a^	2.120 ± 0.010 ^a^	0.181 ± 0.005 ^a^
	Tocopherol	0.49 ± 0.03 ^a^	0.832 ± 0.067 ^a^	5.076 ± 0.339 ^a^	0.236 ± 0.010 ^a^	0.017 ± 0.003 ^a^	2.499 ± 0.028 ^b^	2.133 ± 0.032 ^a^	0.203 ± 0.007 ^a^
Soft Stearin	BHA	0.32 ± 0.04 ^a^	0.617 ± 0.012 ^b^	3.317 ± 0.067 ^b^	0.224 ± 0.009 ^b^	0.018 ± 0.002 ^b^	2.558 ± 0.052 ^a^	2.329 ± 0.022 ^a^	0.176 ± 0.017 ^a^
	Rosemary	0.24 ± 0.03 ^b^	0.621 ± 0.001 ^b^	3.298 ± 0.004 ^b^	0.176 ± 0.016 ^c^	0.018 ± 0.001 ^b^	2.415 ± 0.078 ^b^	1.980 ± 0.002 ^b^	0.183 ± 0.001 ^a^
	Tocopherol	0.31 ± 0.02 ^a^	0.642 ± 0.018 ^a^	3.845 ± 0.099 ^a^	0.282 ± 0.018 ^a^	0.024 ± 0.003 ^a^	2.565 ± 0.067 ^a^	1.999 ± 0.003 ^b^	0.188 ± 0.004 ^a^

Values are the mean of three determinations from two replicate experiments ± standard deviation (*n* = 6), except for the mean values of G-value and total chlorine %, which are the mean of two determinations from two replicate experiments ± standard deviation (*n* = 4). Mean values with different superscript letters in the same column are significantly different at *p* < 0.05.

**Table 3:** Synergistic effects of the antioxidants on the FFA content, specific extinction values and acylglycerol composition in the fats portion extracted from cake baked at 160 °C for 20 min with different shortenings.

Sample	Antioxidants	FFA, %	K_268_, CT	K_232_, CD	FFA/DAG	MAG/DAG	1,3/1,2-DAG
Palm Olein	Rosemary, mg/kg						
	400	0.34 ± 0.13 ^bA^	0.891 ± 0.029 ^bB^	3.453 ± 0.105 ^aB^	0.134 ± 0.023 ^aA^	0.015 ± 0.004 ^aA^	2.367 ± 0.079 ^bA^
	800	0.53 ± 0.04 ^aB^	0.886 ± 0.010 ^bB^	3.487 ± 0.073 ^aB^	0.123 ± 0.020 ^aB^	0.016 ± 0.004 ^aB^	2.564 ± 0.045 ^aA^
	1200	0.42 ± 0.08 ^abA^	0.903 ± 0.001 ^aB^	3.541 ± 0.054 ^aB^	0.108 ± 0.014 ^aB^	0.018 ± 0.005 ^aA^	2.416 ± 0.096 ^aA^
	Tocopherol, mg/kg						
	400	0.39 ± 0.04 ^bA^	1.008 ± 0.028 ^bA^	4.112 ± 0.087 ^cA^	0.148 ± 0.011 ^bA^	0.018 ± 0.003 ^aA^	2.346 ± 0.064 ^bA^
	800	0.63 ± 0.05 ^aA^	0.961 ± 0.001 ^cA^	4.536 ± 0.025 ^bA^	0.223 ± 0.001 ^aA^	0.024 ± 0.005 ^aA^	2.472 ± 0.063 ^aA^
	1200	0.53 ± 0.07 ^aA^	1.085 ± 0.002 ^aA^	4.811 ± 0.008 ^aA^	0.217 ± 0.006 ^aA^	0.019 ± 0.005 ^aA^	2.323 ± 0.050 ^bA^
Soft Stearin	Rosemary, mg/kg						
	400	0.39 ± 0.06 ^bA^	0.728 ± 0.065 ^bB^	3.076 ± 0.045 ^bB^	0.160 ± 0.004 ^cB^	0.019 ± 0.004 ^aA^	2.477 ± 0.077 ^aA^
	800	0.55 ± 0.07 ^aA^	0.740 ± 0.008 ^bB^	2.899 ± 0.059 ^cB^	0.183 ± 0.005 ^bB^	0.028 ± 0.009 ^aA^	2.588 ± 0.034 ^aA^
	1200	0.41 ± 0.06 ^aA^	0.879 ± 0.006 ^aA^	4.171 ± 0.013 ^aA^	0.269 ± 0.011 ^aA^	0.019 ± 0.006 ^aA^	2.420 ± 0.121 ^aA^
	Tocopherol, mg/kg						
	400	0.39 ± 0.03 ^abA^	0.822 ± 0.009 ^aA^	3.712 ± 0.034 ^sA^	0.230 ± 0.032 ^bA^	0.020 ± 0.004 ^aA^	2.512 ± 0.121 ^aA^
	800	0.43 ± 0.04 ^aB^	0.781 ± 0.002 ^bA^	3.678 ± 0.002 ^aA^	0.282 ± 0.013 ^aA^	0.024 ± 0.005 ^aA^	2.523 ± 0.126 ^aA^
	1200	0.35 ± 0.03 ^bA^	0.757 ± 0.003 ^cB^	2.993 ± 0.018 ^aB^	0.280 ± 0.010 ^aA^	0.025 ± 0.001 ^aA^	2.403 ± 0.059 ^aA^

Values are the mean of three determinations from two replicate experiments ± standard deviation (*n* = 6). Mean values with different superscript letters in the same column are significantly different at *p* < 0.05 by ANOVA and the Tukey test. Mean ^AB^ values of the same column for each parameter are significantly different at *p* < 0.05 (comparison between rosemary and tocopherol addition in each sample).
